# Caves as wildlife refuges in degraded landscapes in the Brazilian Amazon

**DOI:** 10.1038/s41598-023-32815-x

**Published:** 2023-04-13

**Authors:** Rafael de Fraga, Valéria Tavares, Matheus Henrique Simões, Xavier Prous, Cesare Girolamo-Neto, Iuri V. Brandi, Guilherme Oliveira, Leonardo C. Trevelin

**Affiliations:** 1 Biodiversity and Ecosystem Services, Instituto Tecnológico Vale, Belém, Pará Brazil; 2 Environmental Licensing and Speleology, Vale S.A., Nova Lima, Minas Gerais Brazil

**Keywords:** Ecology, Community ecology, Conservation biology

## Abstract

Cross-habitat spillover may be the outcome of a process of habitat loss or degradation where the receiving habitat serves as a refuge for organisms. Once surface habitats are lost or degraded, animals can find underground refuge in caves. This paper is focused on testing whether taxonomic order richness inside caves is positively affected by the loss of the native vegetation cover surrounding caves; whether degradation of native vegetation cover predicts cave community composition; and whether there is a pattern of cave community clusters delimited by similarity in the effects of habitat degradation on animal communities. We gathered a comprehensive speleological dataset consisting of occurrence data of thousands of invertebrates and vertebrates sampled in 864 iron caves in the Amazon, to test the effects of both variables measured inside caves and surrounding landscapes on spatial variation in richness and composition of animal communities. We show that caves can work as refuges for the fauna in landscapes where the native vegetation cover surrounding them was degraded, which was evidenced by landcover change increasing the richness of cave communities and clustering caves by similarity in community composition. Therefore, habitat degradation on the surface should be a key variable when characterizing cave ecosystems for conservation prioritization and offset planning. Habitat degradation causing a cross-habitat spillover effect highlights the importance of maintaining the connection between caves by the surface, especially large caves. Our study can help guide industry and stakeholders working on the complex conciliation between land use and biodiversity conservation.

## Introduction

Habitat loss and degradation have been a global concern for biodiversity conservation because they are the leading causes of the ongoing sixth mass extinction^1,2^. Nonetheless, quantifying habitat loss and degradation may not be a trivial exercise because discrete habitats often maintain interdependent ecological dynamics and functions^3^. A notorious case is the challenge of protecting cave biodiversity, inherent in the fact that caves provide resources for rare and micro-endemic species to complete their life cycles while surrounded by landscapes rich in interesting resources for industry, such as wood, minerals and soil for pasture and agriculture^4–6^. Reconciling industrial interests and conservation of cave biodiversity is challenging, but advances have been concentrated in southeastern Amazonia, where the Serra dos Carajás Mineral Province has been widely studied through analyzes of comprehensive speleological databases^7–9^.

Disturbance in epigean landscapes potentially affects the flow of organic sediments and water into caves^10–12^, unbalances cave microclimate and increases erosive processes^13^. Therefore, degrading epigean landscapes around caves can potentially modify the entire trophic structure from which troglobiont (underground exclusive) life cycles depend^6,12^. However, caves are also occupied by a wide variety of trogloxene (aboveground-underground commuters) or troglophilous animals (can maintain populations in the underground, but not necessarily), for which caves are a portion of a broader environmental gradient that can be occupied^14^. Since an immediate response of at least part of the epigean fauna to the habitat loss and degradation may be to flee trying to disperse to alternative habitats^15,16^, it seems plausible the hypothesis that caves in degraded areas may serve as refuges for a variety of animals with different levels of specificity in the occupation of caves as permanent or temporary habitats. The movement of organisms between habitats for both dispersal and foraging (sometimes called “cross-habitat spillover”) has been understood as an essential process affecting wildlife populations and conditioning diversity patterns^17–19^.

Caves usually maintain relatively stable temperature and humidity conditions^20^, although this stability may depend on the geosystem’s integrity. Thus, they can act as critical seasonal refuges for a variety of vertebrates^21,22^ and invertebrates^23^, even in less stressful climate zones such as the Neotropics^24^. However, we are unaware of previous tests to understand if caves may become refuges when habitat quality outside is reduced as a result of the habitat degradation on the surface. Species that do not necessarily complete their life cycles inside caves (non-troglobionts) might occupy these environments seeking shelter from extreme or less stable temperatures and humidity or from predators. Understanding the actual role of caves as wildlife refuges, particularly in anthropogenic landscapes, may refine our perception regarding the impacts of habitat loss and degradation and add crucial information to the planning and execution of offset programs.

In this study, we use a comprehensive speleological database to investigate drivers of animal community richness and composition within caves and to contribute to the ecological bases that guide conservation prioritization and offsetting programs for industry. We considered that the conversion of native land cover in wild landscapes into anthropogenic land use is a process of habitat degradation, and focused on the role of caves located in areas with altered vegetation cover as refuges for animals that usually do not necessarily complete their life cycle inside caves (trogrophilous and trogloxenes). We applied mixed-effects and multivariate linear models to test the hypotheses that (i) non-troglobiont animals occupying caves as refuges in degraded areas increase community richness, but this does not apply to assemblages composed exclusively of troglobiont species; (ii) proportions of areas converted into mining, agriculture, and pasture fields (treated as habitat degradation) are better predictors of community composition of the cave fauna (except for strict troglobionts), than natural characteristics measured inside caves; and (iii) habitat degradation predicts clustering of caves as inferred by similarity in community composition.

## Results

Different variables quantifying habitat degradation were often highly correlated between the different buffers applied (Fig. S3), which suggests that different spatial scales at which the surrounding landscape affects the cave fauna communities are nested. However, the coefficients derived from these variables in GLMMs were considerably higher when extracted from 500-m buffers and tended to become negligible with increasing buffer size (Fig. S4). This finding indicates a negative correlation between the intensity of the habitat degradation effects on the cave fauna communities and the distance from the caves; therefore, we based our conclusions on 500-m buffers.

The most parsimonious GLMM for total order richness retained only mining as a predictor variable (Table S3). It was more parsimonious than a similar model using the sum of all loss categories (ΔAIC 44.4) and a null model (ΔAIC 67.8), suggesting order richness-specific responses to mining. This model explained 53% of the variance in richness when considering caves clustered by regions (R^2^c), although a moderate R^2^m-value (0.1) suggested that the effects of habitat loss varied greatly across regions. This result reflects differences between regions in the proportions of altered areas for mining (ANOVA F_3–869_ = 180, P < 0.0001), although the sizes of the differences varied between paired regions (Fig. S5).

**Figure 1:**
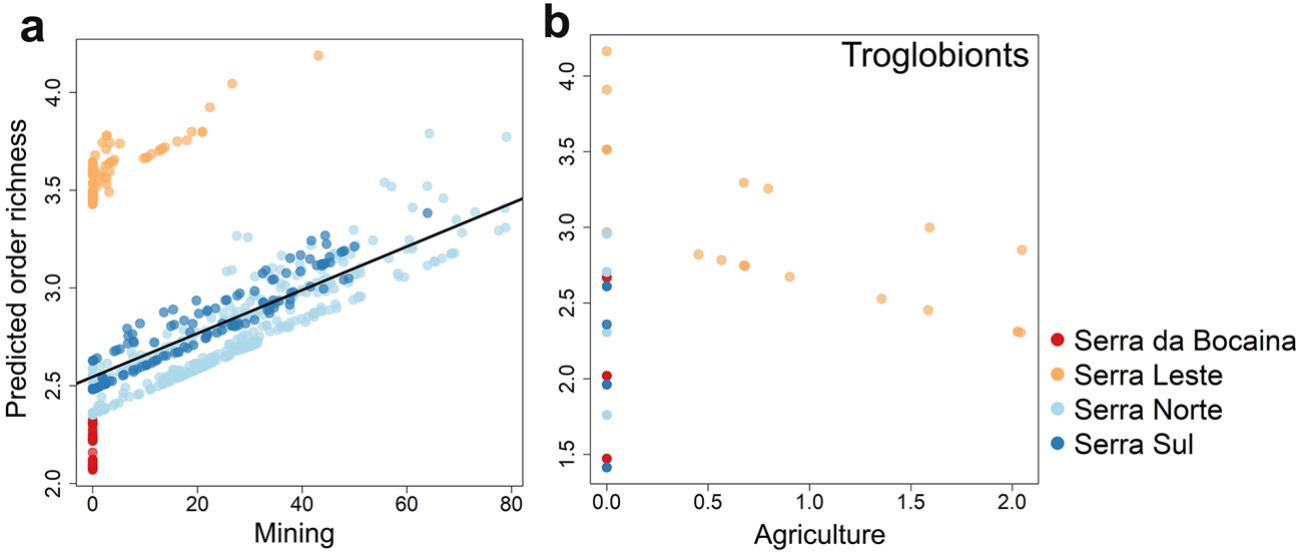
Order richness of total (**a**) and troglobiont communities (**b**) predicted by mixed linear models tested with variables quantifying habitat degradation as fixed-effects predictors and plateaus (colors) of the Carajás region as a random factor.

We found community richness positively affected by mining (z = 6.85, P < 0.0001). Graphically, this finding was particularly associated with a good fit of the model for the more degraded sampling regions, while the currently protected Serra da Bocaina had a relatively poor fit (Fig. 1a). The GLMM for troglobiont richness as a response variable retained only agriculture as a predictor variable after AIC-based selection (Fig. 1b). However, no significant effect of this variable was detected (P = 0.38). Overall, we found that the degradation of native vegetation cover tends to increase the richness of cave fauna communities in the Carajás region, but such a response can only be detected when including non-troglobionts in the communities, as our initial predictions.

**Figure 2:**
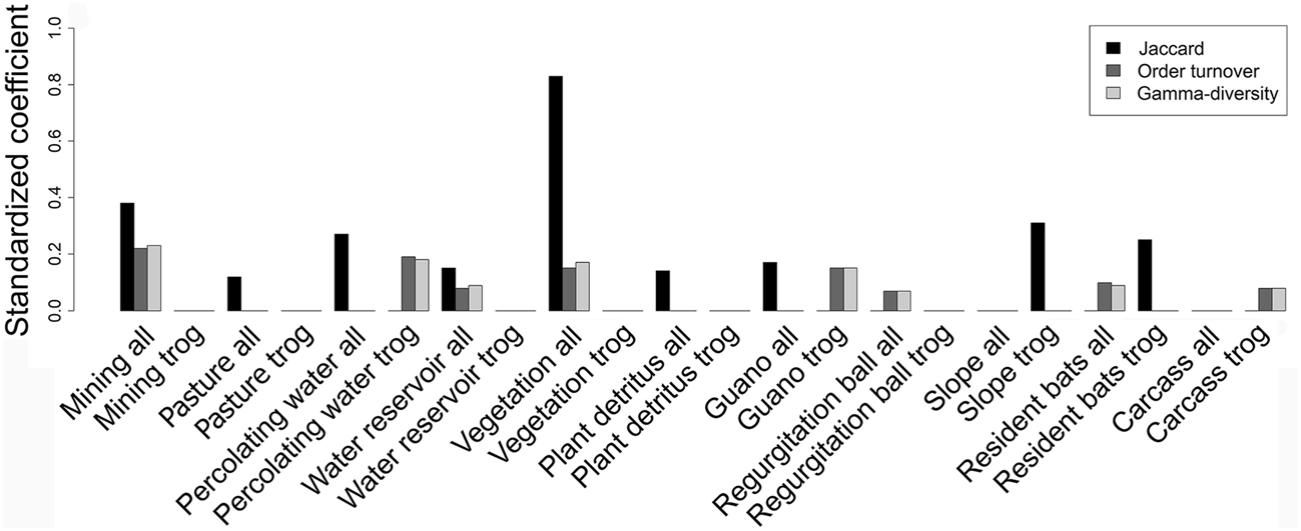
Standardized coefficients derived from mixed linear models tested to identify predictor variables of three community composition metrics (Jaccard dissimilarities, order turnover, local contribution to gamma-diversity) of total cave fauna and troglobionts. The models were fitted with data grouped by four sampling regions in southeastern Amazonia. All = full dataset, trog = troglobionts only.

Considering the composition of complete communities estimated by Jaccard dissimilarities, the most parsimonious GLMM retained mining, pasture, percolating water, water reservoir, vegetation, plant detritus and guano as predictors (Fig. 2). The models accounting for order turnover and local contribution to gamma-diversity estimates as response variables consistently retained mining, resident bats, water reservoir, vegetation and regurgitation balls. All these variables significantly explained the community composition metrics (P < 0.05 in all cases), but mining was the best predictor of community composition in almost all models, except for the Jaccard dissimilarities calculated for the entire fauna community, which was best predicted by a combination of mining and vegetation. Comparing the final models with models using the sum of all categories of habitat degradation revealed that specific sources of habitat loss were more parsimonious than total degradation or null models for all Jaccard dissimilarities (ΔAIC 244; ΔAIC 249.5, respectively), order turnover (ΔAIC 44.8; ΔAIC 54.7) and local contribution to gamma-diversity (ΔAIC 56.9; ΔAIC 66.5).

Considering only troglobionts, the GLMMs identified slope and resident bat populations as predictors of Jaccard dissimilarities, and percolating water, guano and carcass as predictors of both order turnover and local contribution to gamma-diversity estimates. Overall, the GLMMs outputs suggested that variables quantifying habitat degradation have caused more pronounced changes in the composition of cave fauna communities than the other variables measured within or around the caves and were better fitted than null models. Complete results from all GLMMs can be found in Table 1.

**Table 1:** Summary of the results of mixed linear models applied to test the effects of multiple predictor variables on different measures of community composition grouped by region as a random factor.

	Response	Random effects		Fixed effects				
		Variance	SD	Predictor	Estimate	SE	z	P
All taxa	Jaccard	1.62	1.27	Mining	90.02	13.74	6.55	< 0.0001
				Pasture	− 17.24	8.63	− 1.99	0.04
				PW	− 0.53	0.11	− 4.81	< 0.0001
				WR	− 0.34	0.10	− 3.28	0.001
				Veg	− 2.65	0.19	− 13.72	< 0.0001
				Det	0.29	0.09	3.05	0.002
				Guano	− 0.34	0.09	− 3.80	0.0001
	Order turnover	0.26	0.50	Mining	52.92	9.80	5.39	< 0.0001
				Bats	0.39	0.14	2.85	0.004
				WR	− 0.19	0.08	− 2.28	0.02
				Veg	− 0.47	0.11	− 4.18	< 0.0001
				RB	0.51	0.24	2.10	0.03
	Gamma-diversity	0.30	0.54	Mining	53.54	9.68	5.53	< 0.0001
				Bats	0.37	0.14	2.73	0.006
				WR	− 0.20	0.08	− 2.37	0.01
				Veg	− 0.54	0.11	− 4.88	< 0.0001
				RB	0.49	0.24	2.08	0.03
Troglo	Jaccard	2.31	1.52	Slope	0.30	0.07	4.11	< 0.0001
				Bats	− 0.98	0.22	− 4.42	< 0.0001
	Order turnover	0.12	0.65	PW	0.38	0.11	3.36	0.0007
				Guano	0.30	0.09	3.25	0.001
				Carcass	− 0.29	0.15	− 1.89	0.05
	Gamma-diversity	0.43	0.66	PW	0.37	0.11	3.24	0.001
				Guano	0.30	0.09	3.24	0.001
				Carcass	− 0.31	0.15	− 1.98	0.04

Only predictor variables that have had significant effects are shown.

*SD* standard deviation, *SE* standard error, *PW* percolating water, *WR* water reservoir, *Veg* vegetation, *Det* plant detritus, *RB* regurgitation balls, *Troglo* troglobionts.

Moran’s tests revealed that the residuals of some models were positively autocorrelated, which means that the effects of the predictors on community metrics may be more similar among nearby caves than expected by chance. However, Moran’s *I* values obtained for the distance classes with significant spatial autocorrelation were approximately zero, therefore the geographic distance effects on the GLMMs are negligible (Fig. S6).

**Figure 3:**
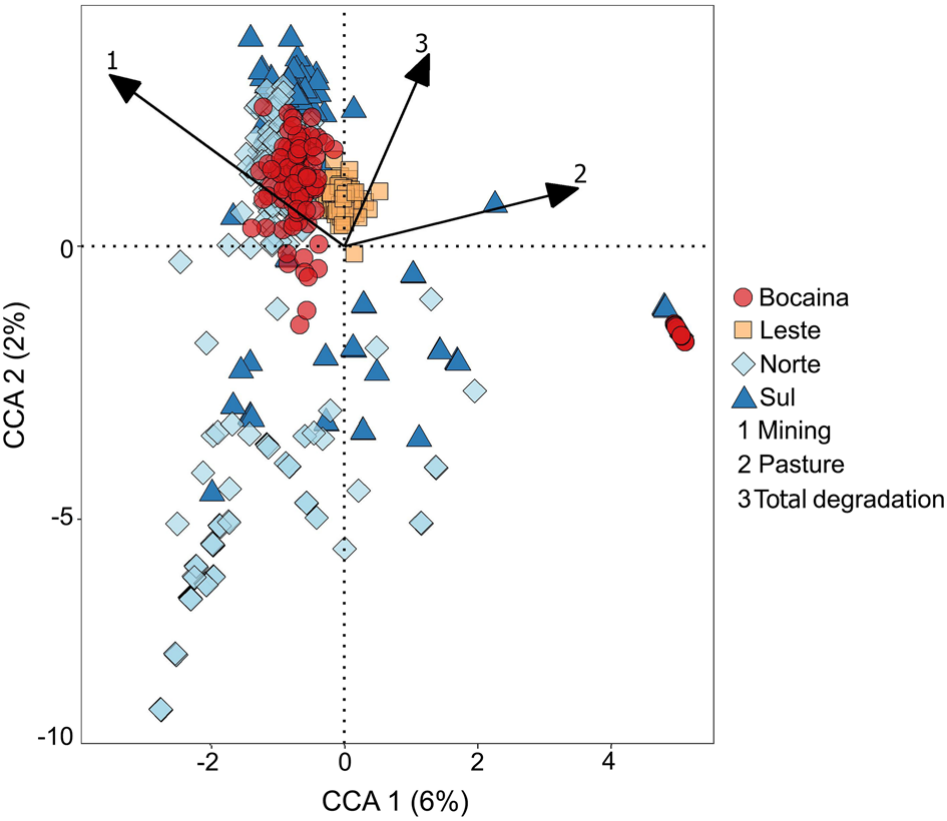
Summarized results of a Canonical Correspondence Analysis (CCA) applied to investigate cave clustering through similarities in the responses of total cave fauna to predictor variables quantifying degradation of native vegetation cover. Values in parentheses are variances captured by each axis.

The first two CCA axes accounted for 6% of the variation in order occurrence, and the cumulative variance explained by these axes of the order-predictor relationships was 21%. A permutation test by axis showed that the relationships between the occurrence of orders and the predictor variables were captured by both axes 1 (F_1–868_ = 29.5, P = 0.001) and 2 (F_1–868_ = 12.2, P = 0.001). A permutation test by terms showed significant effects of mining (F_1–868_ = 20.1, P = 0.001), pasture (F_1–868_ = 18.9, P = 0.001) and the total loss (F_1–868_ = 4.1, P = 0.001). Although observable visual overlap of caves from different regions along the CCA axes (Fig. 3), segregation between all regions was supported by Dunn's tests on at least one of the axes (Table 2). Considering that we modeled the CCA conditioned by the biogeographic aggregation of caves in regions, these results indicate cave aggregation in Carajás generated by similarities in the responses of faunal communities to the habitat degradation.

**Table 2:** Coefficients resulting from Dunn’s tests comparing scores derived from a canonical correspondence analysis (CCA) applied to test grouping of Carajás caves by similarities in the responses of faunal communities to the degradation of native vegetation cover.

Comparison	CCA 1		CCA 2	
	Z	P	Z	P
Bocaina-Leste	− 1.27	0.03	− 1.26	0.41
Bocaina-Norte	15.99	< 0.0001	− 2.69	0.02
Leste-Norte	14.52	< 0.0001	− 0.83	0.40
Bocaina-Sul	8.02	< 0.0001	− 9.20	< 0.0001
Leste-Sul	8.15	< 0.0001	− 6.71	< 0.0001
Norte-Sul	− 6.21	< 0.0001	− 7.79	< 0.0001

## Discussion

We found evidence supporting that habitat degradation surrounding caves in southeastern Amazonia likely promotes the occupation of caves as refuges for epigeic fauna. The conversion of the native land cover into degraded ecosystems triggers community spatial turnover in the landscape, and these disturbed habitats work as a source of organisms in habitat-cross spillover into caves. This pattern only emerges when cave fauna communities are quantified accounting for taxa that do not necessarily complete their life cycle in caves. These findings highlight the key influence of the surrounding landscapes in the occupation of caves and its relevance for biodiversity conservation of subterranean ecosystems^7,9,25^. Furthermore, the integrity of these surrounding landscapes needs to be accounted for when defining the baseline suitability of reference caves used for biodiversity monitoring and cave prioritization protocols that drive environmental licensing and biodiversity offset programs^26^.

According to the Federal Decree Nº 6.640 of the Brazilian Environmental Law (November 7, 2008), the conservation of caves is mainly based on a classification of their relevance regarding community richness and composition in addition to other attributes (e.g. geological, paleontological, scenic, sociocultural). Caves classified as “maximum relevance” are considered a reference for decision-making on offsetting anthropogenic impacts affecting caves (e.g. mining, agriculture). However, our study showed that the richness and composition of cave fauna are not independent of the surrounding landscapes, which draws attention to the suitability of the baseline assumed as a reference. The idea of shifting baseline suitability^27^ highlights that differences between actual conservation states and those perceived through ecological models can bias decision-making on offset planning, evident when considering reference sites that previously suffered from degradation and loss of biodiversity. Suppose caves classified as “maximum relevance” are inserted in landscapes with a history of landcover change. In that case, we suggest that assuming these caves as a reference for conservation should be based on shifting baseline suitability, as ignoring that current conservation state do not necessarily reflect the “original communities” may result in falsely optimistic conservation scenarios^26^.

In caveless tropical rainforests, degradation of native vegetation cover usually decreases the species richness of several groups of plants, vertebrates, and invertebrates^28^. However, degradation in the studied landscapes also increased the order richness of several vertebrates and invertebrates inside caves, suggesting its role as refuge for wildlife, somewhat analogous to microrefugia which have protected biodiversity for thousands of years from fire and other anthropogenic disturbances^29^.

We found no effect of habitat degradation on the predicted distribution of troglobiont taxa. The loss of the native vegetation cover often increases erosion, which can block water and nutrient conduits into caves^30,31^, and change groundwater quality by increased sediment saturation and electrical conductance^32^. Additionally, since changes in landscapes around caves have reduced the quality of underground habitats, one could expect local extinction of troglobionts^6^. The lack of this evidence in our models can be attributed to the fact that in general, intrinsic characteristics of caves were best predictors of the composition of troglobiont communities. In particular, the presence of guano was of high importance, because the increase in the availability of trophic resources provided by guano facilitates the colonization of caves, which may increase not only species richness, but also the occurrence of rare species and even functional and phylogenetic diversity of troglobionts^7,25^.

Although habitat degradation has mostly predicted community composition, some variables measured inside caves also influenced dissimilarities in the occurrence of orders. Caves featuring percolating waters play an essential role in the allochthonous transport of micro and macronutrients or even heavier molecules such as lipids and hydrocarbons^33^. Water reservoirs are essential components for the development of stygofauna and are sources of humidity for the entire cave community. On the other hand, reservoirs can reduce species richness because they reduce the effective ground area of caves or because some species may not tolerate the flood pulses that caves experience in seasonal climate zones^25^. The presence of vegetation, plant remnants and guano also contribute to dissimilarities in the community compositions, increasing the availability of trophic resources and facilitating the colonization of caves and the permanence of non-troglobiont animals for a longer time^20^.

Given our findings, the laws that regulate cave protection in Brazil may also benefit animals that are not strictly dependent on these environments. Our results may be interpreted considering the “spillover effect” concept, described as the benefit of an environmental reserve outside its borders, particularly to the surrounding, unprotected habitats^17^. The spillover effect is mediated by cross-habitat dispersal^19^ represented in our study system by the movements between surface and underground habitats. We argue that a variety of animals will seek resources (e.g. shelter, food, water) within caves as degradation reduces the quality of external landscapes. Habitats with higher densities of organisms usually have strong spillover effects (e.g.^34^).

Considering the positive relationship between the degradation of the native vegetation cover and cave richness and that a positive relationship between cave richness and cave area has been previously observed for the Carajás caves^25^, large caves inserted in areas heavily degraded are likely to suffer from a stronger spillover effect. The benefits of the spillover effects may vary between species with different dispersal abilities^17^, so future studies could perform analyses similar to those presented here separately by taxonomic group (e.g. classes). Detecting these effects in the Carajás caves have relevant implications for cave and landscape conservation and management, and indicates the need to connect or keep the connections between large caves by implementing corridors of conserved or recovered native vegetation.

It is extremely important to highlight that the positive effects between surface landscape degradation caused by mining and the richness of cave fauna should not be an argument for encouraging non-monitored mining activities. Instead, our results have key implications for the conservation of cave biodiversity, particularly in matrices of degraded habitats. We have shown that the degradation of the native vegetation cover around caves interferes with the distribution of the overall biodiversity, because it increases species richness in caves and heterogeneity in the spatial distribution of fauna communities. This initially implies that caves should not be studied in isolation from the landscapes in which they are located. Furthermore, habitat degradation should be considered as part of biological monitoring and ecological modeling studies focused on cave conservation, use, licensing for mineral exploitation and offset planning. Based on our results we suggest that the dispersal of animals triggered by habitat degradation generates spillover effects that must be considered for estimating targets for biodiversity conservation.

We expect our findings to apply to researchers, estimates of impact and monitoring assessments of industry and stakeholders acting on the complex reconciliation between land use and biodiversity conservation. Such application could be refined by future studies focused on accessing ecological effects of the cross-habitat spillover on the original fauna, such as competition and niche displacement^35^, and estimating for how long these effects can be detectable or even if they are permanent. Further framing the time scale in which the process we describe on this study takes place is of the essence, as presently observed patterns might be governed by additional time-lag effects in faunal movements or historical differences in governance of the landscapes surrounding caves^36,37^. Substantial conversion of native landcover due to mining in our study region started 30 years before sampling of the present study took place^38^, enough to promote the clear pattern of habitat spill-over depicted here, although the effects of differential deforestation rates between sites remain to be accessed.

## Materials and methods

### Study region

**Figure 4:**
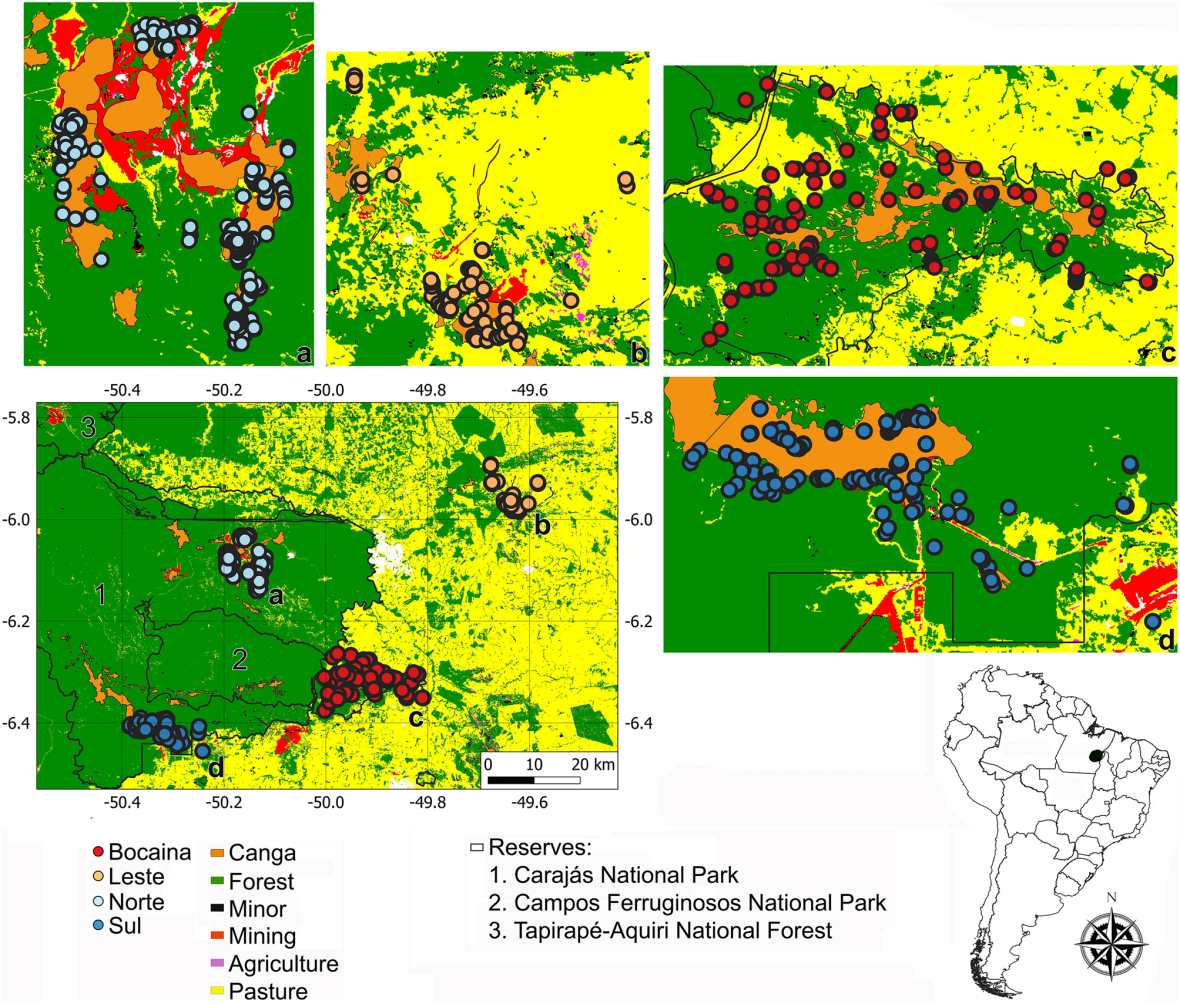
Location of the study (black ellipse in the map). In the detail the numbers represent the three reserves in the area (Carajás and Tapirapé-Aquiri National forests, and Campos Ferruginosos National Park) and caves within each plateau are represented by different colors (Serra da Bocaina, Serra Leste, Serra Norte, and Serra Sul).

The region known as Serra dos Carajás (Fig. 4), located in southeastern Amazonia (centroid coordinates 49° 55′ 0″ W, 6° 8′ 49″ S), harbors a complex subterranean ecosystem with more than 1500 natural iron caves^39^ and one of the largest iron reserves and mining enterprises in the world^40^. The relief of this region is characterized by plateaus that can exceed 840 m in elevation, which are connected by primary and secondary lowland rainforests with varying levels of understory density, rich in vines and palm trees, and high biomass^41^. At the top of the plateaus are patches of forest and savannas regionally called cangas, which are predominantly covered by grasses and shrubs, including several endemic species^42^. Most caves are associated with the scarp lines of these plateaus, thus in the transition between forest patches and canga formations^43^. The regional climate is marked by a dry season between June and September and a rainy season between October and May. The average annual precipitation is usually around 2000 mm, and the average temperature is 25 °C, with slight annual variation, although the daily variation may exceed 10 °C during the rainy season^44^.

Four relatively isolated plateaus can be readily identified in the study area, each differing in the numbers of caves recorded, the sizes of the canga patches, their occupancy history and levels of reserve protection. Serra da Bocaina, with 204 sampled caves, has not been explored for mining and is presently well protected by the Campos Ferruginosos National Park. However, in previous decades the surroundings of caves have been converted into pasture fields and minor areas of other land uses (e.g. aquaculture, planted forests). On this plateau, caves are mainly located in transition zones between forests and cangas and reach up to 3.5 km^2^. Serra Leste contains severely modified landscapes by pasture and mining zones and is the only sampled plateau not currently protected by reserves. On this plateau, we sampled 117 caves in highly fragmented forests and transition zones with canga patches reaching up to 2.8 km^2^. Serra Norte has been severely modified by mining and pasture fields, although its insertion in the Carajás National Forest guarantees the protection of the immediate surroundings of caves (up to 250 m from the entrance). On this plateau, we sampled 375 caves in forest areas and transition zones with canga patches, reaching up to 2.8 km^2^. Serra Sul was inserted into well-preserved lowland forests near a few small degradation sources during the sampling, although it has recently been altered by mining. On this plateau we sampled 177 caves in forest areas and transition zones with large canga patches, covering up to 4.2 km^2^. Since these plateaus are isolated by geomorphological discontinuities and physiographic differences^45^, we assumed them in this study as management units.

### Data collection

Our database was compiled from data collected by several teams of speleologists that sampled the invertebrate and vertebrate fauna from 873 caves in Serra dos Carajás region in non-consecutive surveys from 2010 to 2014. These original datasets have mainly been collected for environmental impact assessments, licensing and monitoring programs related to iron ore mining activities. Part of this data has been previously published (e.g.^7,8,25^). Sampling methods were based on parameters established by the Brazilian legislation (Federal Decree 6640/2008 and Normative Instruction MMA 02/2009), requiring surveys through each cave’s full extension. Data collection consisted of two sampling events in each cave, comprising the dry and the rainy seasons. Taxonomic assignments were evaluated by taxonomists expertized in different groups, who identified specimens at the finest possible taxonomic level. However, several remarkably diverse invertebrate groups were represented mainly by undescribed species. In this case, specimens were assigned to morphotypes.

We excluded taxa for which taxonomic identification or assignment to a morphotype was impossible (e.g. unidentified invertebrate larvae) and compiled all the available data into a single dataset consisting of 23 classes (five vertebrates, 18 invertebrates) distributed in 106 orders (11 vertebrates, and 95 invertebrates), 542 families (33 vertebrates, 509 invertebrates), and 3553 species (121 vertebrates, 3432 invertebrates). Complete data about the sampled taxa can be found in Supporting Information Table S1. We coded community data as binary, presence or absence of taxa per cave, instead of abundance, assuming that the variation in abundance across caves could be due to seasonality^9^.

We also gathered datasets with relevant information about the caves, which compose most of the predictive variables that we used in the inferential models. Data were collected following a standard protocol that included the geographic coordinates of the cave entrance, elevation, slope, cave area, occurrence of resident groups of bats, percolating water, water reservoir, vegetation, plant detritus, roots, guano, feces of non-bat animals, regurgitation balls (undigested material accumulated and regurgitated by predators), and carcasses. Further details on data collection and motivations for the use of these variables can be checked in Table S2.

We also generated data on the environments surrounding caves to quantify the relevance of the caves for biodiversity conservation considering the landscape. We used geographic coordinates to obtain average Height Above the Nearest Drainage (HAND) values in 500-m buffers around the caves. HAND was calculated based on an algorithm considering the unidirectional flow to determine the preferred path of water on the ground to the nearest drain. The values are computed from the topography from the Digital Elevation Model (MDE) of SRTM—The Shuttle Radar Topography Mission^46^. We downloaded a raster with a resolution of 1 km in the AMBDATA^47^ repository and extracted values per cave using the raster R-package^48^.

Since native ecosystems around the caves have been converted into degraded habitats to native fauna (e.g. mining pits with no vegetation cover, agriculture and pasture grass fields), we assumed proportional conversion of areas into anthropogenic zones as a measure of habitat degradation. To quantify it, we used Landsat-based land use maps available in the MapBiomas Brasil repository^49^. To account for time-lag effects (delay in the responses of fauna communities to the habitat degradation), we used maps provided for the year before the surveys. Once the plateaus were sampled in distinct years, our data on habitat degradation is a compilation of values extracted from multiple maps. The original map classes can be found in Table S3, but we reclassified them as 1 representing native vegetation, 2 for other types of alteration summing minor areas, such as planted forest, urbanization, and aquaculture (treated here as minor loss), 3 for mining, 4 for agriculture, 5 for pasture, and the sum of categories 2–5 as total habitat degradation. Since we had no basis for deciding a priori the size of the altered area around caves that potentially affects cave fauna, we calculated proportions of pixels representing habitat degradation in 500, 1000, 1500 and 2000 m buffers. However, we based our conclusions only on the buffer size that best predicted community composition. We also compared models with specific habitat degradation categories and models with total degradation (sum of all categories). This was a practical approach to evaluating whether community responses to habitat degradation are specific. All predictor variables used in the ecological models are summarized in Table 3, and complete datasets can be found in the Supplementary material S1.

**Table 3:** Summary of variables used to predict animal richness of taxonomic orders and composition from 873 iron caves in southeastern Amazonia.

Variable type	Variable	Min	Max	Mean	SD
Inside caves, continuous	Elevation	201	842	554.1	153.1
	Slope	0.1	38.20	3.73	4.2
	Area	5.00	4224	127.9	231.5
Landscape, continuous	HAND	0	595.8	329.5	165.5
	Minor loss	0	19.79	2.74	3.99
	Mines	0	79	14.92	18.09
	Agriculture	0	2.04	0.02	0.17
	Pasture	0	40.12	20.58	30.07

Variable type	Variable	Frequency (%)			
Inside caves, discrete	Resident bats	7			
	Percolating water	51.8			
	Water reservoir	22.9			
	Vegetation	89.5			
	Plant detritus	62.8			
	Roots	96.9			
	Guano	48			
	Feces (non-bats)	38.8			
	Regurgitation balls	1.8			
	Carcasses	8.2			

Minimum (Min), maximum (Max), mean and standard deviation (SD) values are shown for the continuous variables and frequencies for categorical variables (percentage of pixels in which a variable has value 1 meaning presence). HAND is height above the nearest drainage. Habitat loss (minor, mines, agriculture and pasture) is shown as percentage of pixels in 500-m buffers.

### Community-level analysis

Our dataset comprises thousands of records of vertebrates and invertebrates. Since our sampling is mainly composed of macroinvertebrates for which there are many taxonomic gaps and undescribed taxa, 19% (N = 676) of the sampled taxa could not be identified at lower levels than order, while 81% of sampled taxa were identified at family, genus or species level. To consider genus, species or family occurrence data would imply ruling out almost half of caves with less than five occurrences, which would bias estimates of paired dissimilarities in community composition. This was specifically demonstrated in our study by a non-metric multidimensional scaling (NMDS) based on Jaccard dissimilarities in taxa occurrence. Using orders as taxonomic resolution yielded less loss of information by compressing dimensionalities and lower global Stress (Fig. S1). Once we wanted to reach the greatest levels of comparability between caves and plateaus, and the best possible representation of multidimensional communities, we performed all analyses at the order level. Although some studies have suggested that lower taxonomic levels than order are required for ecological models (e.g.^50^), this does not seem consensual. There are studies showing that using orders may be sensitive enough for ecological models to efficiently capture biotic complementarities between samples^51–53^, and even generate multivariate clusters that are more reliable compared to lower taxonomic levels^54^. We hereafter refer to the complete set of sampled orders as communities assuming that they are “groups of interacting populations occurring together in space”^55^.

We used mixed-effects linear models (GLMMs) implemented in the glmmTMB R-package^56^ to assess whether richness of taxonomic orders is positively affected by habitat degradation for the full dataset and, in a separate analysis, including only records of troglobionts. These models were built using the number of orders as the response variable, and the percentages of minor loss, mining, agriculture and pasture as predictor variables of fixed effects. As random factors, we used the sampling plateau (Serra Norte, Serra Sul, Serra Leste and Serra da Bocaina) to control for spatial autocorrelation effects in the residuals and also cave area categories (the area was classified into five size levels to reduce the effects of species-area relationships: < 100 m^2^, 100–500 m^2^, 600–900 m^2^, and > 1000 m^2^). We implemented negative binomial distribution models with quadratic parameterization and Broyden-Fletcher-Goldfarb-Shanno algorithm, namely the BFGS optimizer^56^. Finally, we used the MuMIn R-package^57^ to obtain values of marginal R^2^ (R^2^m), representing the variance explained by the fixed effects, and conditional R^2^ (R^2^c), which represents the conditional explained variance associated with fixed effects plus the random effects.

We used the same predictor variables and random factors to test whether different types of habitat degradation are better predictors of three different estimates of the community composition than other variables measured within caves or in the surrounding landscapes. We used the vegan R-package^58^ to quantify pairwise Jaccard dissimilarities in taxonomic order occurrence summarized by Principal Coordinate Analysis (PCoA); the betapart R-package^59^ to isolate the turnover component of beta-diversity based on Jaccard dissimilarities summarized as a PCoA axis; and the contribdiv function of the vegan R-package^58^ to estimate the contribution of each cave to the regional diversity (gamma diversity).

Chiroptera data could be problematic, because the presence of resident bats and guano were used as predictor variables in the GLMMs, and this could confuse causal inferences (e.g. presence of guano depends on bats, and both are consequence of bat fauna composition). However, removing Chiroptera from the dataset had negligible effects on the community richness and composition estimates (Fig. S2), so that GLMMs results were not qualitatively altered.

Because all the community composition estimates were beta-distributed, as we checked using fitdistrplus R-package^60^, we implemented GLMMs in the glmmTMB R-package^56^ configured for the beta family. We found the most parsimonious model in all GLMMs tested for order richness and community composition by comparing AIC values across all possible combinations of predictor variables. We also scaled all continuous predictor variables to mean 0 and standard deviation 1, and ensured that none of the final models was configured with highly correlated predictors (> 70%). To graphically compare the coefficients derived from the GLMMs among predictors, we standardized them using the parameters R-package^61^.

To test spatial autocorrelation in the GLMM-derived residuals we used Moran’s tests implemented in the pgirmess R-package^62^. Results are shown by correlograms with ten classes of geographic distance between caves.

We applied partial Canonical Correspondence Analyses (CCA) to discriminate clusters formed by similar community responses to the habitat degradation. This enabled us to test for levels of biotic redundancy among caves and plateaus by overlapping caves along multivariate axes and summarizing the responses of the faunal communities to the habitat degradation. Since the sampling design is characterized by the aggregation of caves on plateaus separated by lowland forest corridors, clusters formed by similarities in community composition could reflect biogeographic differences between plateaus. To overcome this potential bias, we included a resistance surface based on elevation as a conditioning matrix in the CCA. From an elevation raster obtained from the public repository Ambdata^47^, we applied the costDistance function from the gdistance R-package^63^. This function uses Dijkstra's algorithm to calculate the least-cost path between all paired coordinates, starting from a transition layer connecting eight cells around each geographic coordinate of the caves. The output is a matrix of paired dissimilarities, which we summarized as a principal coordinate axis. We included the cost of altitude for connectivity between caves to reduce the effects of biogeographic isolation on dissimilarities in the community composition, so the CCA should show cave clustering mediated by the degradation of the native vegetation cover. We performed the CCA using the vegan R-package^58^, plotted the outputs using ggplot2^64^, and tested the significance of axes and predictors using a test of 999 permutations of canonical axes, following^65^. To test whether caves clustered consistently along regions sampled, we used Dunn's nonparametric tests implemented in the FSA R-package^66^, with the scores for sites derived from the first and second axes of the CCA separately as response variables, and region as a factor.

## Supplementary Information

Supplementary Information 1.

Supplementary Information 2.

Supplementary Information 3.

## Data Availability

All data generated or analysed during this study are included in this published article in the Supplementary Material S1.
