# Supplementary material for: Caves as wildlife refuges in degraded landscapes in the Brazilian Amazon
Source: Sci Rep. 2023 Apr 13;13:6055. doi: 10.1038/s41598-023-32815-x (PMC10102069; doi:10.1038/s41598-023-32815-x)

**Supplementary Information**

**Caves as wildlife refuges in degraded landscapes in the Brazilian Amazon**

**Fraga R, Tavares V, Simões MH, Prous X, Girolamo-Neto C, Brandi IV, Oliveira G, Trevelin LC**

**Table S1.** Summary of taxa sampled in 876 caves in the Carajás region, eastern Amazonia.

| **Class** | **Nº orders** | **Nº families** | **Nº species** |
| --- | --- | --- | --- |
| Acanthocephala | 1 | 1 | 1 |
| Actinopterygii | 1 | 1 | 1 |
| Amphibia | 1 | 7 | 30 |
| Annelida | 2 | 5 | 31 |
| Arachnida | 23 | 134 | 829 |
| Aves | 3 | 3 | 5 |
| Bivalvia | 1 | 1 | 1 |
| Chilopoda | 6 | 14 | 104 |
| Chromadorea | 1 | 1 | 1 |
| Diplopoda | 8 | 24 | 118 |
| Entognatha | 5 | 28 | 139 |
| Gastropoda | 5 | 10 | 53 |
| Gordioida | 1 | 1 | 1 |
| Insecta | 30 | 262 | 2016 |
| Malacostraca | 2 | 13 | 76 |
| Mammalia | 4 | 12 | 69 |
| Nemathelminthes | 3 | 3 | 8 |
| Pauropoda | 1 | 1 | 2 |
| Reptilia | 2 | 10 | 16 |
| Rhabditophora | 1 | 1 | 1 |
| Symphyla | 1 | 4 | 21 |
| Turbellaria | 3 | 5 | 25 |
| Udeonychophora | 1 | 1 | 5 |
| **Total** | 106 | 542 | 3553 |

**Table S2.** Predictor variables used in mixed-effects generalized linear models applied to investigate spatial structure of cave fauna communities, respective justifications, and relevant bibliographic references.

| **Predictor** | **Method** | **Justification** |
| --- | --- | --- |
| Elevation | Measured with GPS at the cave entrance. | Changes in biodiversity metrics in response to elevation is one of the best documented patterns in ecology, and some authors consider it as ubiquitous as the latitudinal diversity gradient ^[1]^. |
| Slope | The steepness length of cave terrain was measured using Autocad (m). | Influences the amount of resources that are carried or washed into caves, by gravity ^[2]^. |
| Area | Calculated in Autocad (m³) based on a laser-scanned model. | Following the premises of species/area relationships, larger caves have more species, the cave area increases wealth and alters the composition of communities ^[2]^. |
| Resident bats | Three 20-m equidistant observers verified the presence of colonies with adults, juveniles, and females with puppies. | Bats regulate several ecological processes in caves, which affect nutrient cycles ^[3]^. |
| Percolating water | Three equidistant 20 m observers checked the entire extent of the cave. | Promotes allochthonous transport of micro and macronutrients, and heavier molecules such as lipids and hydrocarbons ^[4]^. |
| Water reservoir | Three equidistant 20 m observers checked the entire extent of the cave. | Essential component for the development of stygofauna and sources of humidity for the entire cave community. It may reduce species richness by reducing the effective ground area of caves or because some species may not tolerate the flood pulses that caves experience in seasonal climate zones ^[5]^. |
| Plant detritus | Three equidistant 20 m observers checked the entire extent of the cave. | Increases the availability of trophic resources, facilitating the colonization of caves and the permanence of non-troglobiont animals for a longer time ^[3,6]^. |
| Roots | Three equidistant 20 m observers checked the entire extent of the cave. | Increases the availability of trophic resources, facilitating the colonization of caves and the permanence of non-troglobiont animals for a longer time ^[3,6]^. |
| Guano | Three equidistant 20 m observers checked the entire extent of the cave. | Increases in the availability of trophic resources, facilitating the colonization of caves, which may increase species richness, occurrence of rare species, functional and phylogenetic diversity ^[2,5]^. |
| Feces (non-bats) | Three equidistant 20 m observers checked the entire extent of the cave. | Increases the availability of trophic resources, facilitating the colonization of caves and the permanence of non-troglobiont animals for a longer time ^[3,6]^. |
| Regurgitation balls | Three equidistant 20 m observers checked the entire extent of the cave. | Increases the availability of trophic resources, facilitating the colonization of caves and the permanence of non-troglobiont animals for a longer time ^[3,6]^. |
| Carcasses | Three equidistant 20 m observers checked the entire extent of the cave. | Increases the availability of trophic resources, facilitating the colonization of caves and the permanence of non-troglobiont animals for a longer time ^[3,6]^. |
| Animal occurrence | Animals were sampled by active search, by three 20-m equidistant observers, who searched all the substrates available throughout the extent of the cave. The sampling time depended on the cave area. Specimens were collected with tweezers (invertebrates) and dip nets (bats). Invertebrates were allocated in 70% alcohol for taxonomic identification in the laboratory by experts. Vertebrates were photographed, accommodated in cotton bags, and sent for identification by experts. | Fauna communities show ecological responses that are very useful for decision making on cave conservation in areas under anthropogenic pressure ^[2,5,7]^. |

References:

1. Sanders N J, Rahbek C. 2012. The patterns and causes of elevational diversity gradients. Ecography 35: 1–3.

2. Jaffé R, Prous X, Calux A, Gastauer M, Nicacio G, Zampaulo R, Souza-Filho P WM, Oliveira G, Brandi IV, Siqueira JO (2018) Conserving relics from ancient underground worlds: assessing the influence of cave and landscape features on obligate iron cave dwellers from the Eastern Amazon. PeerJ 6:e4531

3. Culver DC, Pipan T. 2019. The biology of caves and other subterranean habitats. Oxford University Press, New York.

4. Li Y, Šimůnek J, Wang S, Yuan J, Zhang W. 2017. Modeling of soil water regime and water balance in a transplanted rice field experiment with reduced irrigation. Water 9: 248–262.

5. Jaffé R, Prous X, Zampaulo R, Giannini TC, Imperatriz-Fonseca VL, Maurity C, Oliveira G, Brandi IV, Siqueira JO (2016) Reconciling mining with the conservation of cave biodiversity: a quantitative baseline to help establish conservation priorities. PLoS ONE 11(12):e0168348

6. White WB, Culver DC. 2012. Encyclopedia of caves. Waltham: Academic Press.

7. Trevelin LC, Gastauer M, Prous X, Nicácio G, Zampaulo R, Brandi I, Oliveira G, Siqueira JO, Jaffé R (2019) Biodiversity surrogates in Amazonian iron cave ecosystems. Ecol Indic 101(5):813–820

**Table S3.** Original land use categories assigned by MapBiomas (mapbiomas.org), and reclassifications used in this study to measure deforestation around caves in the Carajás region, southeastern Amazonia.

| **Original category** | **Reclassification** |
| --- | --- |
| Planted forest | Minor degradation |
| Urbanized area |  |
| Other non-vegetated areas |  |
| Aquaculture |  |
| Soy | Agriculture |
| Sugar cane |  |
| Rice |  |
| Coffee |  |
| Citrus |  |
| Other temporary crops |  |
| Other perennial crops |  |
| Pasture | Pasture |
| Mining | Mining |

**Fig. S1.** Stressplots showing relations between dissimilarities in the composition of fauna communities observed between paired plots, and those estimated by multidimensional non-metric scaling (NMDS). NMDS models were configured with animals identified as species, families and orders.

**
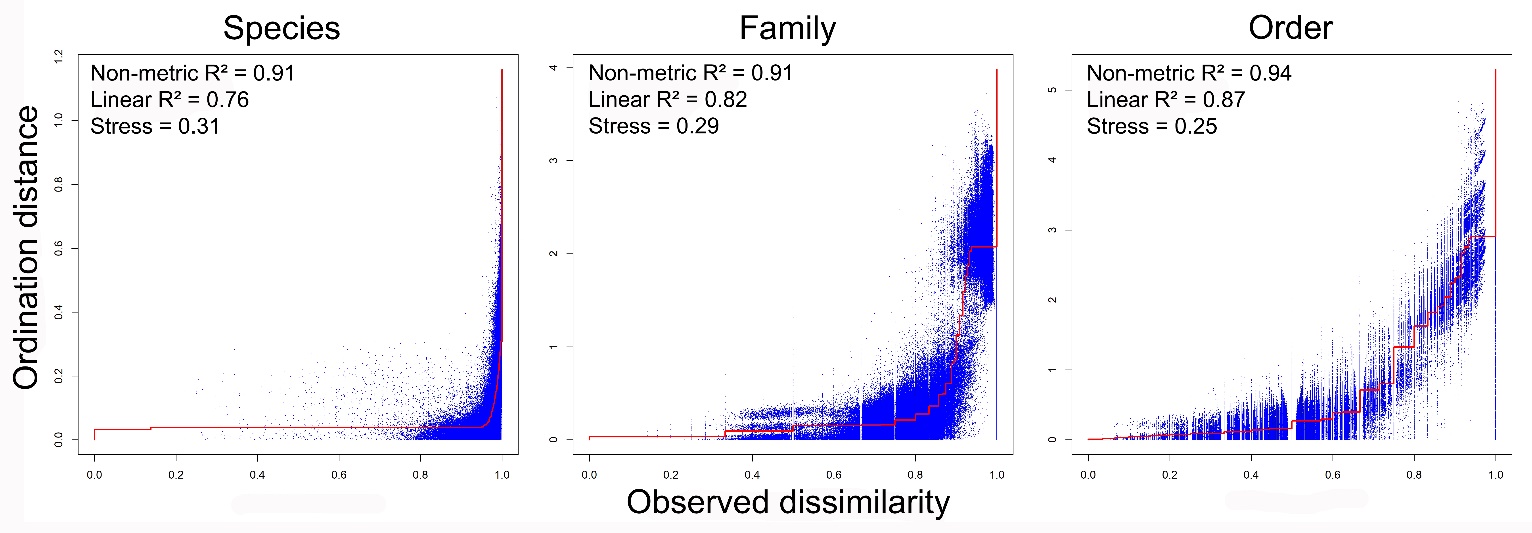
**

**Fig S2.** Correlations between order richness (A) and composition (B) with and without bats in the dataset. Composition was estimated by Jaccard dissimilarities in the occurrence of orders between paired caves, summarized as a principal coordinate axis.

**
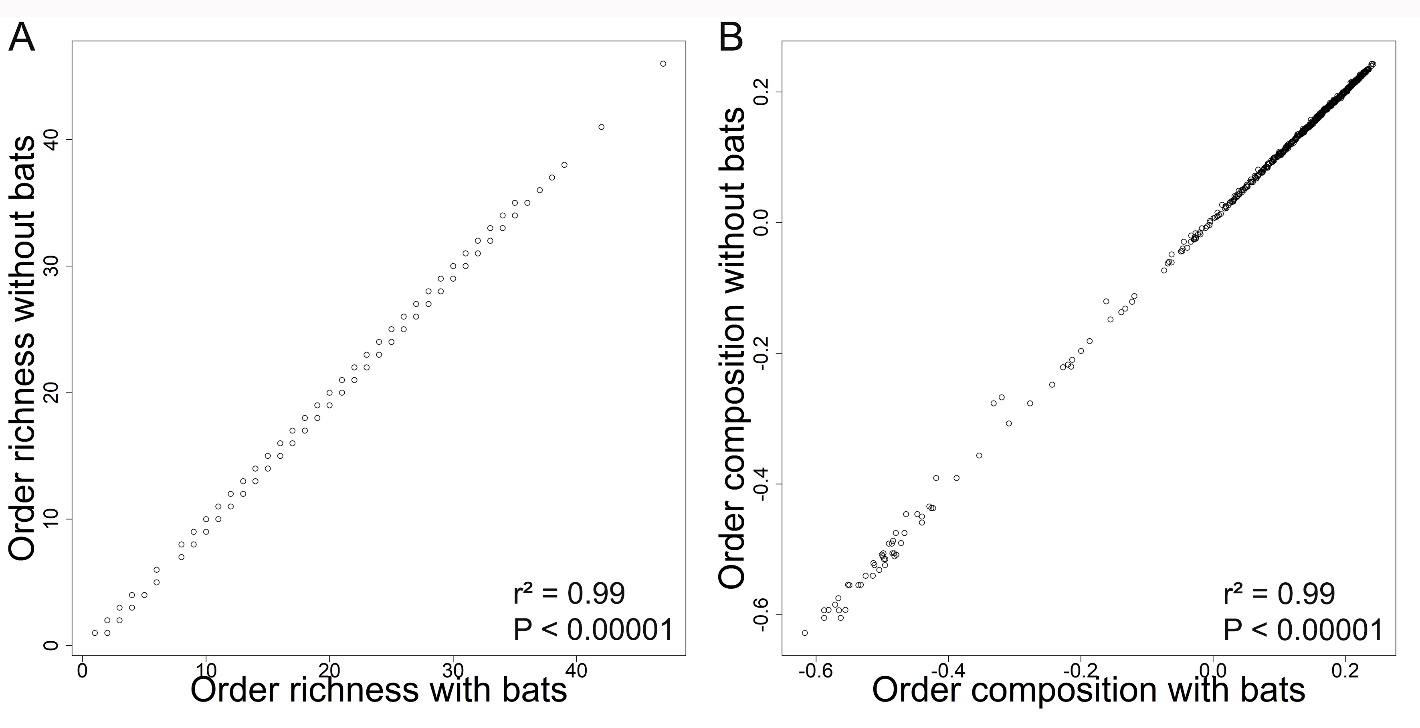
**

**
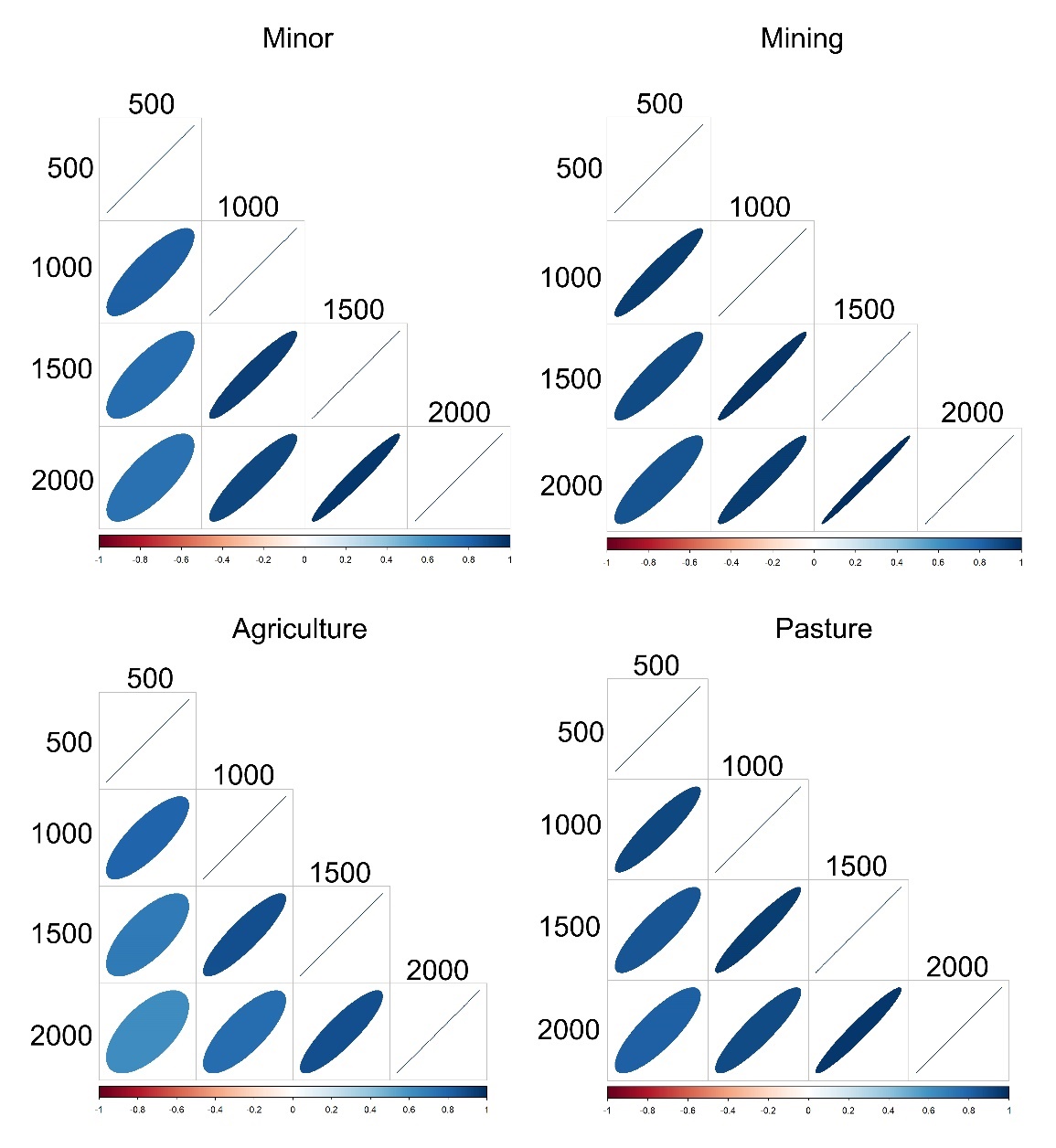
Fig. S3.** Correlations between variables quantifying habitat degradation extracted from 500, 1000, 1500 and 2000 m buffers around the Carajás caves. The intensity and direction of the correlations are coded by the thickness of the ellipses and shades of blue (stronger shades show greater positive correlations).

**Fig. S4.** Coefficients of linear mixed models (GLMMs) testing the effects of predictor variables on Jaccard dissimilarities (A), order turnover (B) and local contribution to gamma diversity (C) of cave fauna in Carajás cavities, compared between different buffer sizes (500, 1000, 1500 and 2000 m) from which values of habitat loss were extracted. PW = percolating water, WR = water reservoir, Veg = vegetation.

**
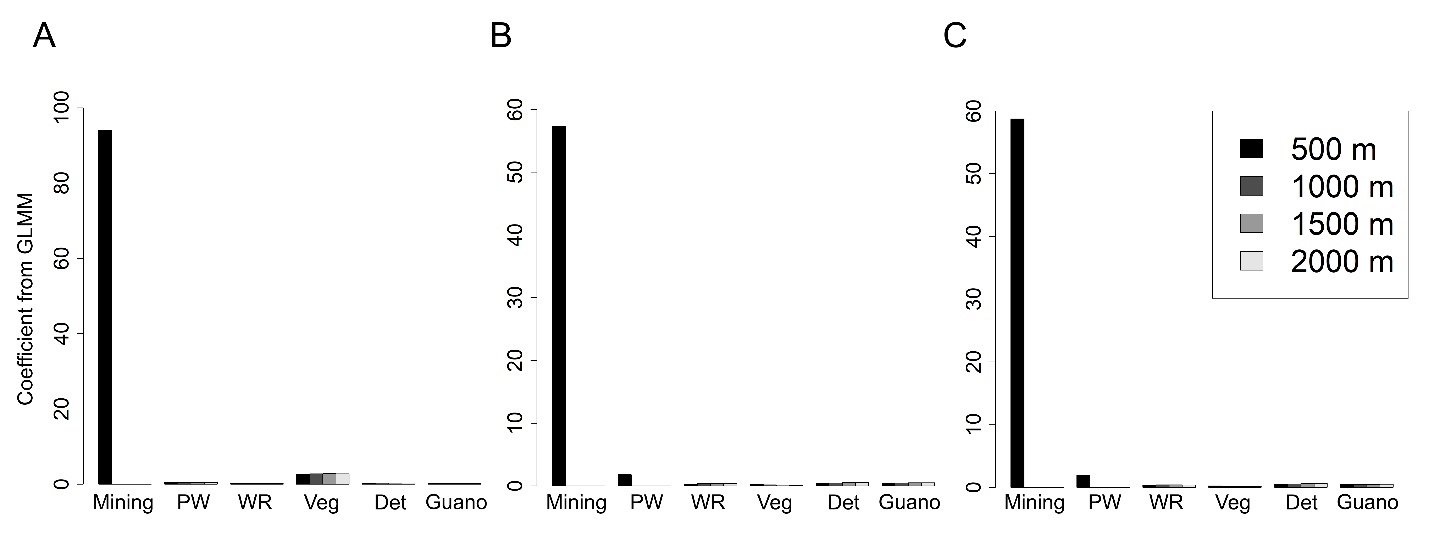
**


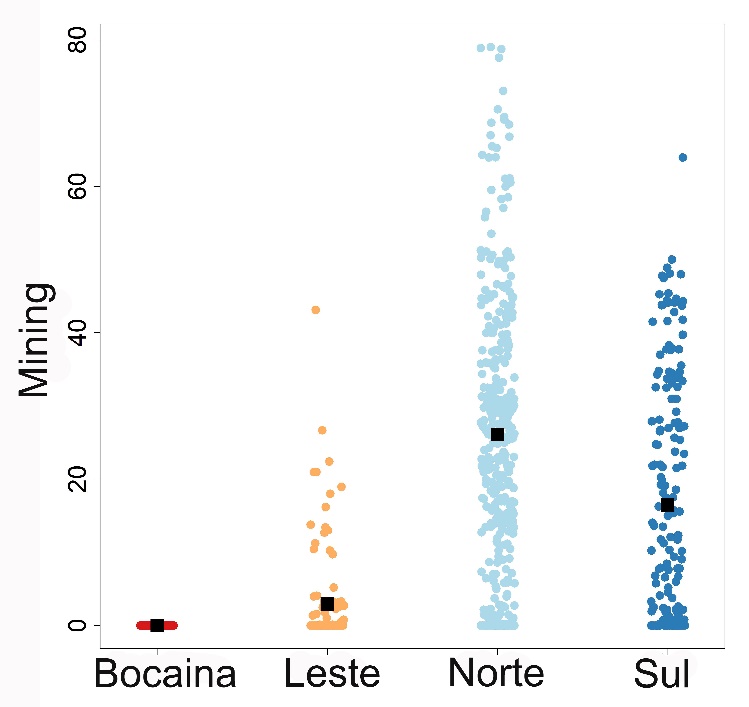
**Fig. S5.** Comparison of proportional habitat degradation in 500 m around cavities in Carajás. Degradation is shown as relating to mining. Black squares are means.

**Fig S6.** Correlograms showing spatial autocorrelation in residuals derived from generalized mixed-effects linear models. Red circles are cases where the null hypothesis of the Moran´s *I* test was rejected at P < 0.05.


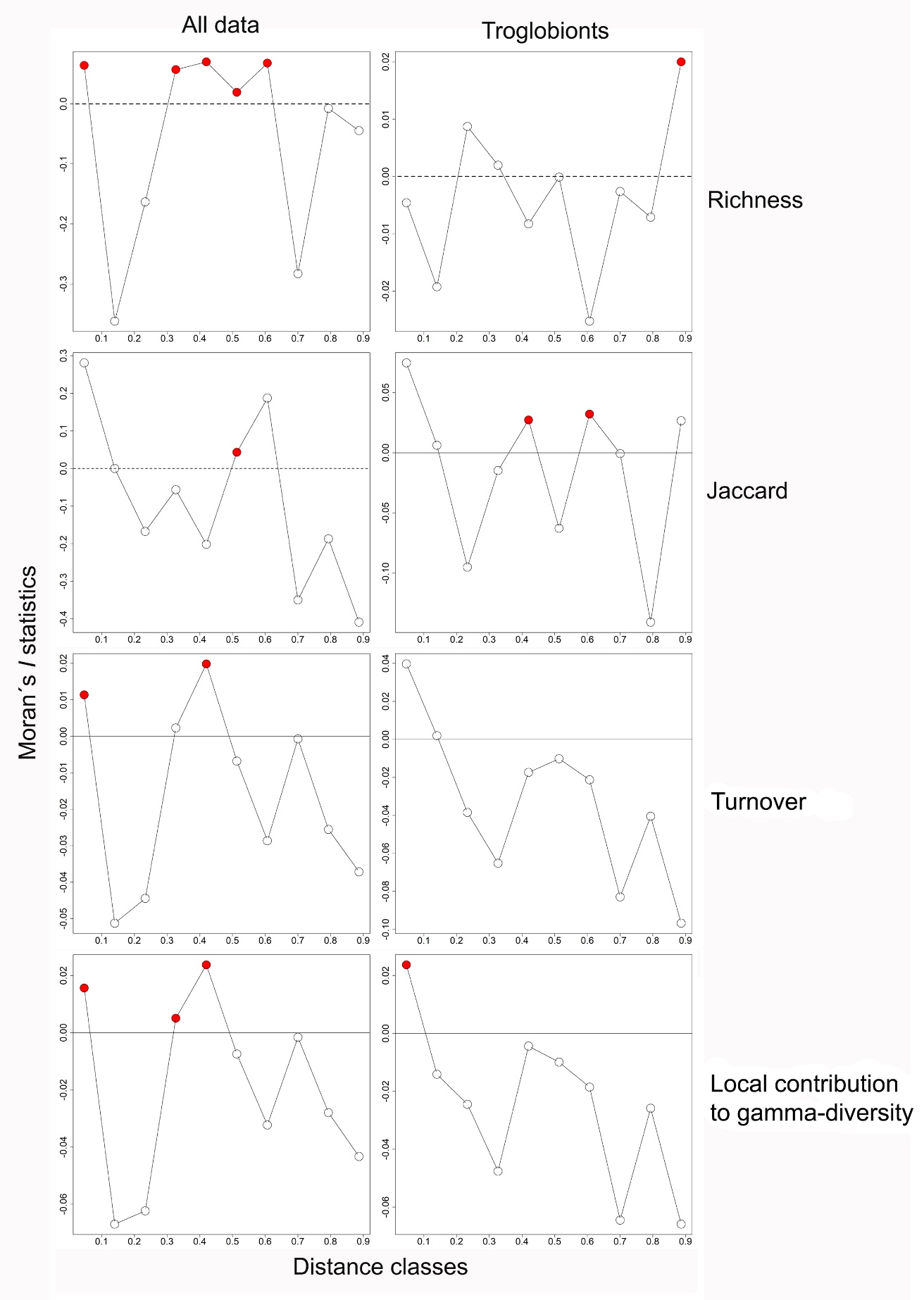

Supplement: Supplementary file 1 — Supplementary Information 1. [file 41598_2023_32815_MOESM1_ESM.docx]
